# Newborn Screening for Mitochondrial Carnitine-Acylcarnitine Cycle Disorders in Zhejiang Province, China

**DOI:** 10.3389/fgene.2022.823687

**Published:** 2022-03-14

**Authors:** Duo Zhou, Yi Cheng, Xiaoshan Yin, Haixia Miao, Zhenzhen Hu, Jianbin Yang, Yu Zhang, Benqing Wu, Xinwen Huang

**Affiliations:** ^1^ Department of Genetics and Metabolism, Children’s Hospital, Zhejiang University School of Medicine, National Clinical Research Center for Child Health, National Regional Medical Center for Children, Hangzhou, China; ^2^ School of Health in Social Science, The University of Edinburg, Edinburg, United Kingdom; ^3^ Zhejiang Bosheng Biotechnology Co, Ltd, Hangzhou, China; ^4^ Children’s Medical Center, University of Chinese Academy of Science - Shenzhen Hospital, Shenzhen, China

**Keywords:** mitochondrial carnitine-acylcarnitine cycle disorders, carnitine palmitoyl transferase 2 deficiency, carnitine palmitoyltransferase 2 deficiency, carnitine-acylcarnitine translocase deficiency, newborn screening

## Abstract

**Background:** Disorders of mitochondrial carnitine–acylcarnitine cycle is a heterogeneous group of hereditary diseases of mitochondrial β-oxidation of fatty acids tested in NBS program in Zhejiang province, China. Large-scale studies reporting disorders of mitochondrial carnitine–acylcarnitine cycle among Chinese population in NBS are limited. The aim of this study was to explain the incidence and biochemical, clinical, and genetic characteristics of disorders of mitochondrial carnitine–acylcarnitine cycle in NBS.

**Methods:** From January 2009 to June 2021, 4,070,375 newborns were screened by tandem mass spectrometry. Newborns with elevated C0 levels and/or C0/(C16 + C18) ratios were identified as having CPT1D, whereas those with decreased C0 levels and/or C0/(C16 + C18) ratios and/or elevated C12-C18:1 level were identified as having CPT2D or CACTD. Suspected positive patients were further subjected to genetic analysis. All confirmed patients received biochemical and nutritional treatment, as well as follow-up sessions.

**Results:** Overall, 20 patients (12 with CPT1D, 4 with CPT2D, and 4 with CACTD) with disorders of mitochondrial carnitine–acylcarnitine cycle were diagnosed by NBS. The overall incidence of these disorders was one in 203,518 newborns. In toal, 11 patients with CPT1D exhibited increased C0 levels and C0/(C16 + C18) ratios. In all patients of CPT2D, all long chain acyl-carnitines levels were elevated except for case 14 having normal C12 levels. In all patients with CACTD, all long chain acyl-carnitines levels were elevated except for case 17 having normal C12, C18, and C18:1 levels. Most patients with CPT1D were asymptomatic. Overall, two of 4 patients with CPT2D did not present any clinical symptom, but other two patients died. In 4 cases with CACTD, the disease was onset after birth, and 75% patients died. In total, 14 distinct mutations were identified in CPT1A gene, of which 11 were novel and c.1910C > A (p.S637T), c.740C > T (p.P247L), and c.1328T > C (p.L443P) were the most common mutations. Overall, 3 novel mutations were identified in CPT2 gene, and the most frequent mutation was c.1711C > A (p.P571T). The most common variant in SLC25A20 gene was c.199-10T > G.

**Conclusion:** Disorders of mitochondrial carnitine–acylcarnitine cycle can be detected by NBS, and the combined incidence of these disorders in newborns was rare in Zhejiang province, China. Most patients presented typical acylcarnitine profiles. Most patients with CPT1D presented normal growth and development, whereas those with CPT2D/CACTD exhibited a high mortality rate. Several novel CPT1A and CPT2 variants were identified, which expanded the variant spectrum.

## Introduction

Carnitine cycle is essential for transporting long-chain fatty acids into the mitochondrial matrix for β-oxidation (Watkins et al., 2007). Mitochondrial carnitine–acylcarnitine cycle is an important component of carnitine cycle, which consists of carnitine palmitoyl-transferases 1 (CPT1), carnitine palmitoyl-transferases 2 (CPT2), and the transporter protein carnitine-acylcarnitine translocase (CACT), which can uptake fatty acyl-CoAs across the mitochondrial membrane (Lodhi and Semenkovich, 2014). First, CPT1 located on the outer mitochondrial membrane converts the long-chain acyl-CoAs to their acylcarnitine equivalents. Further, acyl-carnitines are transported into the mitochondrial matrix by CACT. In the end, acyl-carnitine is converted back to the acyl-CoA species and carnitine by CPT2 associated with the inner mitochondrial membrane (McGarry and Brown, 1997). CPT1 deficiency (CPT1D), CPT2 deficiency (CPT2D), and CACT deficiency (CACTD) are rare autosomal inherited recessive disorders, and the causative genes are CPT1A, CPT2, and SLC25A20, respectively (Wieser et al., 2003; Collins et al., 2010; Indiveri et al., 2011). Differences in the age of onset and clinical manifestations were observed in these disorders (Houten and Wanders, 2010). Late onset forms may present muscular symptoms such as myalgia, myoglobinuria, and muscle weakness during adolescence or adulthood. The condition can be triggered by fasting, illnesses, or high energy demand resulting in hypoketotic hypoglycaemia, hepatomegaly, cardiomyopathy, liver dysfunction, seizures, and sudden death at an early age. Disorders of mitochondrial carnitine–acylcarnitine cycle present different mortality and morbidity rates. In particular, CACTD was characterised with high mortality (Baruteau et al., 2014; Houten et al., 2016). Early detection and medical interventions can prevent individuals from metabolic decompensation and improve prognosis (Maguolo et al., 2020).

Infants with these diseases presented characteristic acylcarnitine profile are detectable by using tandem mass spectrometry (MS/MS), which has been widely implemented in NBS. With the application of MS/MS, the diagnosis of these disorders through newborn screening has become easier (Wilcken et al., 2003). The combined incidence of disorders of mitochondrial carnitine–acylcarnitine cycle was thought to be rare, which is estimated to be 1:186,833 in mainland China, 1:250,000 to 1:666,6671 in Australia, Germany, and <1:189,136 in the United States (Lindner et al., 2010; Deng et al., 2020). Disorders of mitochondrial carnitine–acylcarnitine cycle have great phenotypic variability and molecular heterogeneity. Herein, we present our over 12-years experience of NBS for the diagnosis and treatment of disorders of mitochondrial carnitine–acylcarnitine cycle in Zhejiang Province, China. The aim of this study was to determine the incidence and biochemical, clinical, and genetic characteristics of these disorders by NBS. With this study, the awareness about these rare disorders can be improved, and further implications of treatment can be considered.

## Materials and Methods

### Study Population

From January 2009 to June 2021, 4,070,375 newborns were screened by MS/MS at Children’s Hospital, Zhejiang University School of Medicine. Among 4,070,375 participants who were recruited in this study, 3,784,837 were normal-term infants, 276,482 were premature infants, and 9,056 were post-term infants. The ratio of male to female was 1.1:1. The Ethical Committee of Children’s Hospital, Zhejiang University School of Medicine approved this research. The information sheet with consent information containing the explanation on the aim of this study was sent to participants and their parents and was signed by participants’ parents.

### NBS for Disorders of Mitochondrial Carnitine–Acylcarnitine Cycle

Dried blood spot (DBS) samples were randomly collected between 3 and 7 days after birth and delivered through cold-chain transportation to the NBS centre of Children’s Hospital, Zhejiang University School of Medicine within 3 days. Acylcarnitine profiles were assessed at our centre using NeoBase™ MS/MS reagent kits (PekinElmer, United States) according to the manufacturers’ protocol. Newborns with elevated C0 levels and/or C0/(C16 + C18) ratios were identified to have CPT1D. Newborns with decreased C0 levels and/or C0/(C16 + C18) ratios and/or elevated C12-C18:1 level were identified to have CPT2D or CACTD. Newborns were subjected to further confirmatory tests if the initial screening value exceeded the diagnostic cut-off. Newborns were subjected to a repeated test if the initial screening value was equal to or exceeding the screening cut-off. If the second screening results were still positive, confirmatory detection tests were performed, including biochemical laboratory tests and genetic analysis.

### Genetic Testing

Genomic DNA was extracted from patients. Genetic testing was performed by Genetic Diagnostic Laboratory at Children’s Hospital, Zhejiang University School of Medicine (Hangzhou, Zhejiang, China). The target next-generation sequencing (NGS) was used on the probands with a target sequencing panel covering 306 known genes associated with inherited metabolic disorders. All potentially pathogenic mutations identified by NGS were further confirmed via Sanger sequencing. PolyPhen-2, PROVEAN, and Mutation Taster were used to predict the pathogenicity of the missense variants. The Structure stability analysis of novel missense variants were performed by Chimera version 1.15. The protein PDB number was entered in the Chimera software, and the three-level structure of protein was obtained. We selected the amino acid sequence and optimised the structure to find out whether the mutant amino acid triggers the atoms clash and contact. The three-dimensional structure of the protein was constructed after mutation, and the effect of mutation on protein stability was analysed.

### Diagnosis and Follow-Up

Diagnosis was assessed by metabolic disease specialists based on the patients’ free carnitine and acylcarnitine levels, genetic variations, as well as additional clinical symptoms. All confirmed patients were followed up in our hospital. Dietary guidance including more frequent feeding, avoiding fasting, limiting fat intake, and appropriate supplementation of l-carnitine were applied for the confirmed patients. The follow-up time was once every 3–6 months if the confirmed patients presented stable performances. Patients were regarded as lost to follow-up if the follow-up time exceeded over 3 months and the patient still did not return to the clinical centre. The monitoring items included physical and biochemical examinations; levels of free carnitine and acylcarnitine, blood glucose, lactic acid, and blood ammonia; and blood routine test.

## Results

### Incidences Determined From NBS

In this study, 4,070,375 newborns were screened and 20 patients (12 with CPT1D, 4 with CPT2D, and 4 with CACTD) were identified, with a total incidence of one in 203,518 live births. The incidence of CPT1D, CPT2D, and CACTD was 1:339,197, 1:1,017,593, 1:1,017,593, respectively. Among all the disorders of mitochondrial carnitine–acylcarnitine cycle, CPT1D was the most prevalent. There were two pairs of monozygotic twins among patients with CPT1D.

### Initial Screening Results of Acylcarnitine Levels

Overall, 11 patients (91.7%) with CPT1D had increased C0 levels and C0/(C16 + C18) ratios. C0 levels and C0/(C16 + C18) ratios of case 12 were normal in initial screening accompanied with lower C12-C18:1 level. In these patients, the average C0 level was 125.1 ± 61.68 μmol/L (range: 14.84–240.28 μmol/L) and C0/(C16 + C18) was 206.3 ± 278.5 (range: 25.59–1,007.27).

Overall, two of 4 patients with CPT2D had low C0 levels, and those of other two patients were normal. All long chain acyl-carnitines levels were elevated except in case 14 with normal C12 levels. The C14, C16, C16:1, C18, and C18:1 levels were 1.59 ± 0.25, 19.36 ± 2.33, 1.69 ± 0.18, 4.28 ± 0.74, and 6.67 ± 0.89 μmol/L, respectively. The ratios of long-chain acyl-carnitines to C0 were decreased and those to C2 and C3 were increased in all cases (C16 + C18:1)/C2, C14/C3, C16/C3, C16:1/C3, C18/C3, C18:1/C3, (C16 + C18:1)/C3, and C0/(C16 + C18) were, 3.6 ± 2.7, 8.2 ± 2.55, 89.36 ± 53.08, 8.39 ± 5.25, 23.89 ± 14.61, 33.41 ± 21.43, 122.8 ± 73.72, and 0.51 ± 0.29, respectively.

In total, three patients with CACTD had decreased C0 levels. ALL C0/(C16+C18) ratios were decreased. All long-chain acyl-carnitines levels were elevated except in case 17 with normal C12, C18, and C18:1 levels. The C14, C16, and C16:1 levels were 1.33 ± 0.54, 18.97 ± 8.5, and 1.5 ± 0.7 μmol/L, respectively. All the ratios of long-chain acyl-carnitines to C2 and C3 were increased except in case 17 with normal C18/C3 level. The (C16 + C18:1)/C2, C14/C3, C16/C3, C16:1/C3, C18/C3, C18:1/C3, (C16 + C18:1)/C3, and C0/(C16 + C18) were 2.84 ± 1.71, 1.43 ± 0.45, 20,52 ± 7.99, 1.63 ± 0.51, 3.57 ± 1.72, 6.42 ± 4.09, 26.9 ± 11.33, and 0.38 ± 0.13, respectively.

C14/C3, C16/C3, C16:1/C3, C18/C3, C18:1/C3, and (C16 + C18:1)/C3 ratios were higher in patients with CPT2D when compared with those with CACTD. The differences were statistically significant (*p* = 0.038, 0.042, 0.042, 0.032, 0.048, and 0.042, respectively) (Table 1).

**TABLE 1:** Initial screening results of acylcarnitine levels in Mitochondrial Carnitine-Acylcarnitine Cycle Disorders.

Acylcarnitine index	C0	C2	C3	C12	C14	C16	C16:1	C18	C18:1	C0/(C16 + C18)	—	—	—	—	—	—	—
CPT1D	—	—	—	—	—	—	—	—	—	—	—	—	—	—	—	—	—
1	119.08	11.01	0.89	0.03	0.03	0.29	0.02	0.16	0.16	237.33	—	—	—	—	—	—	—
2	110.8	13.1	1.28	0.03	0.01	0.07	0.01	0.04	0.03	1,007.27	—	—	—	—	—	—	—
3	156	37.83	2.01	0.05	0.08	0.86	0.06	0.27	0.3	138.01	—	—	—	—	—	—	—
4	66.9	23.3	1.19	0.06	0.09	0.32	0.04	0.18	0.64	133.8	—	—	—	—	—	—	—
5	117.45	35.71	1.86	0.08	0.11	0.37	0.05	0.17	0.55	217.5	—	—	—	—	—	—	—
6	165.49	36.16	2.3	0.14	0.15	0.76	0.09	0.19	0.2	174.2	—	—	—	—	—	—	—
7	240.28	33.83	3.21	0.08	0.08	0.59	0.05	0.17	0.25	316.16	—	—	—	—	—	—	—
8	131.13	15.47	2.41	0.01	0.02	0.11	0.01	0.1	0.05	624.43	—	—	—	—	—	—	—
9	199.04	28.7	2.42	0.06	0.09	0.79	0.05	0.23	0.25	195.14	—	—	—	—	—	—	—
10	172.35	23.03	0.83	0.02	0.03	0.2	0.01	0.12	0.06	538.59	—	—	—	—	—	—	—
11	72.46	31.68	3.66	0.05	0.09	0.37	0.04	0.15	0.56	139.35	—	—	—	—	—	—	—
12	14.84	4.67	0.46	0.01	0.03	0.4	0.02	0.18	0.27	25.59	—	—	—	—	—	—	—
Acylcarnitine index	C0	C2	C3	C12	C14	C16	C16:1	C18	C18:1	C0/(C16 + C18)	(C16 + C18:1)/C2	C14/C3	C16/C3	C16:1/C3	C18/C3	C18:1/C3	(C16 + C18:1)/C3
CPT2D																	
13	25.34	23.83	0.19	0.72	1.85	17.87	1.63	5.92	7.78	0.91	1.08	9.74	94.05	8.58	31.16	40.95	135
14	5.86	11.47	1.68	0.23	1.24	22.31	1.54	4.24	5.9	0.22	2.46	0.74	13.28	0.92	2.52	3.51	16.79
15	12.41	3.27	0.13	0.59	1.61	17.15	1.63	4.54	7.02	0.57	7.39	12.38	131.92	12.54	34.92	54.00	185.92
16	9.25	7.33	0.17	0.47	1.69	20.09	1.96	4.58	5.98	0.37	3.56	9.94	118.18	11.53	26.94	35.18	153.35
CACTD																	
17	5.56	11.11	0.95	0.18	0.73	8.8	0.9	1.07	2.01	0.56	0.97	0.77	9.26	0.95	1.13	2.12	11.38
18	7.24	7.96	0.63	0.89	1.08	15.23	1	3.07	7.43	0.4	2.85	1.71	24.17	1.59	4.87	11.79	35.97
19	6.86	6	0.88	0.4	1.52	24.36	1.9	4.12	6.15	0.24	5.09	1.73	27.68	2.16	4.68	6.99	34.67
20	10.29	12.83	1.31	0.65	1.99	27.49	2.39	4.73	6.27	0.32	2.63	1.52	20.98	1.82	3.61	4.79	25.77

Cutoff value: C0 10.28–54.24 μmol/L; C2 3–50 μmol/L; C3 0.43–3.8 μmol/L C12 0.03–0.28 μmol/L; C12:1 0.01–0.23 μmol/L; C14 0.07–0.40 μmol/L; C14:1 0.03–0.26 μmol/L; C16 0.49–6.00 μmol/L; C16:1 0.02–0.49 μmol/L; C18 0.24–1.90 μmol/L; C18:1 0.38–2.92 μmol/L; C0/(C16 + C18) 2.76–35.1 (C16 + C18:1)/C2 0.11–0.54; C14/C3; C16/C3 0.35–4.92; C16:1/C3 0.01–0.34; C18/C3 0.6–1.69 (C16 + C18:1)/C3 0.71–7.21.

### Initial Clinical Manifestations

The follow-up period of these patients lasted from 7 days to 4 years. Among 12 patients with CPT1D, 10 patients were asymptomatic. Case 2 presented clinical symptoms after birth, including hyperammonaemia and metabolic acidosis and passed away at 18 days-point. Follow-up data of case 1 were not available.

Patients 13 to 16 were identified as having CPT2D. Patient 13 died suddenly at 8 months at home with no available data. Patient 14 presented with poor feeding, poor response, and hypoglycaemia. Her condition deteriorated rapidly, and she died at 10 days after birth. No clinical symptoms were found in patients 15 and 16; however, patient 15 had incomplete right bundle branch block in ECG, and patient 16 presented transient increase in alanine aminotransferase (ALT; 102 U/L) level.

Patients 17 to 20 were identified to have CACTD. All the patients had disease onset after birth. They presented feeding dysfunction, poor response, hypoglycaemia [1.9–3.2 mmol/L (normal range 3.6–6.1 mmol/L)], acidosis [1.8–17 mmol/L (normal range 0.5–1.6 mmol/L)], and hyperammonaemia [52–226 μmol/L (normal range 9–30 μmol/L)]. After diet management, l-carnitine supplementation, and acidosis correction, the clinical presentations and biochemical results of case 17 became normal, and the data were good after 15-months follow-up. case 18 developed hepatic impairment, hepatomegaly, and cardiac hypertrophy; further, the parents stopped the treatment, and the infant died at the age of 2 months. The condition of cases 19 and 20 deteriorated rapidly, and they died at 7 days after birth (Table 2).

**TABLE 2:** Clinical presentation, biochemical, and genetic investigation of confirmed patients with CPT1D, CPT2D, CACTD.

Patient no	Gender	Age^a^	Genotype		Onset	Clinical presentation
			Allele 1	Allele 2		
CPT1D						
1	Female	3M	c.740C>T	c.1328T > C	ND	ND
2	Female	18D	c.1817G>A	c.1817G>A	After birth	died at 18D hyperammonemia, metabolic acidosis
3	Female	4Y3M	c.979C > T	c.1336G > A	None	asymptomatic
4	Female	5M	c.1910C > A	c.1910C > A	None	asymptomatic
5	Female	5M	c.1910C > A	c.1910C > A	None	asymptomatic
6	Male	3Y9M	c.281+1G>A	c.956G>T	None	asymptomatic
7	Male	3Y	c.2246G > A	c.1131G > C	None	asymptomatic
8	Male	3Y2M	c.577delC	c.740C>T	None	asymptomatic
9	Male	2Y4M	c.205G > A	c.740C>T	None	asymptomatic
10	Female	2Y4M	c.1328T > C	c.1328T > C	None	asymptomatic
11	Male	2Y8M	c.2125G > A	c.1295C > T	None	asymptomatic
12	Male	2Y8M	c.2125G > A	c.1295C > T	None	asymptomatic
CPT2D						
13	Female	8M	c.988dupT	c.1711C > A	8M	died at 8M
14	Female	10D	c.1711C > A	c.520G > A	2D	died at 10D metabolic acidosis, hypoglycemia
15	Male	2Y3M	c.895-896insGGGCAGAGCTC	c.1711C > A	None	asymptomatic incomplete right bundle branch block
16	Male	6M	c.1054T > C	c.1301T > C	None	asymptomatic impaired liver function
CACTD						
17	Male	1Y3M	c.1A>G	c.199-10T>G	After birth	acidosis, hypoglycemia, hyperammonemia, survive
18	Female	3M	c.270delC	c.804delG	After birth	died at 2M metabolic acidosis, impaired liver function, hepatomegaly, cardiac hypertrophy, hyperammonemia
19	Male	7D	c.199-10T>G	c.199-10T>G	After birth	died at 7D acidosis, hypoglycemia, hyperammonemia
20	Female	7D	c.199-10T>G	c.199-10T>G	After birth	died at 7D acidosis, hypoglycemia, hyperammonemia

a Y: years; M: months; D: days; Age^a^: age at last follow-up; ND: no data; *CPT1A* gene for CPTID; *CPT2* gene for CPTIID; *SLC25A20* gene for CACTD.

### Genetic Findings

A total of 14 distinct mutations were identified in CPT1A gene; 85.72% (12/14) were missense, 7.14% (1/14) were deletion, and 7.14% (1/14) were spicing variants. Overall, 11 of these CPT1A variants were novel, and the other three were previously reported. The most frequent mutation was c.1910C > A (p.S637T), with an allelic frequency of 16.67% (4/24). The relatively frequent mutations were c.740C > T (p.P247L) and c.1328T > C (p.L443P), and each of mutation was identified three times, with an allelic frequency of 12.5% (3/24). c.2125G > A (p.G709R) and c.1295C > T (p.P432L) were each identified twice. The remaining six mutations were identified only once.

A total of six distinct mutations were identified in CPT2 gene. Among which, 83.33% (4/6) were missense, and 16.67% (2/6) were frameshift variants. Overall, three of these CPT2 variants were novel. The most frequent mutation was c.1711C > A (p.P571T), with an allelic frequency of 37.5% (3/8). Each of the remaining five mutations were identified only once.

There were 4 distinct mutations in the SLC25A20 gene. All the mutations were previously reported. The most common variant was c.199-10T > G, which accounted for 62.6% (5/8). Each of the remaining three mutations was identified only once.

**FIGURE 1:**
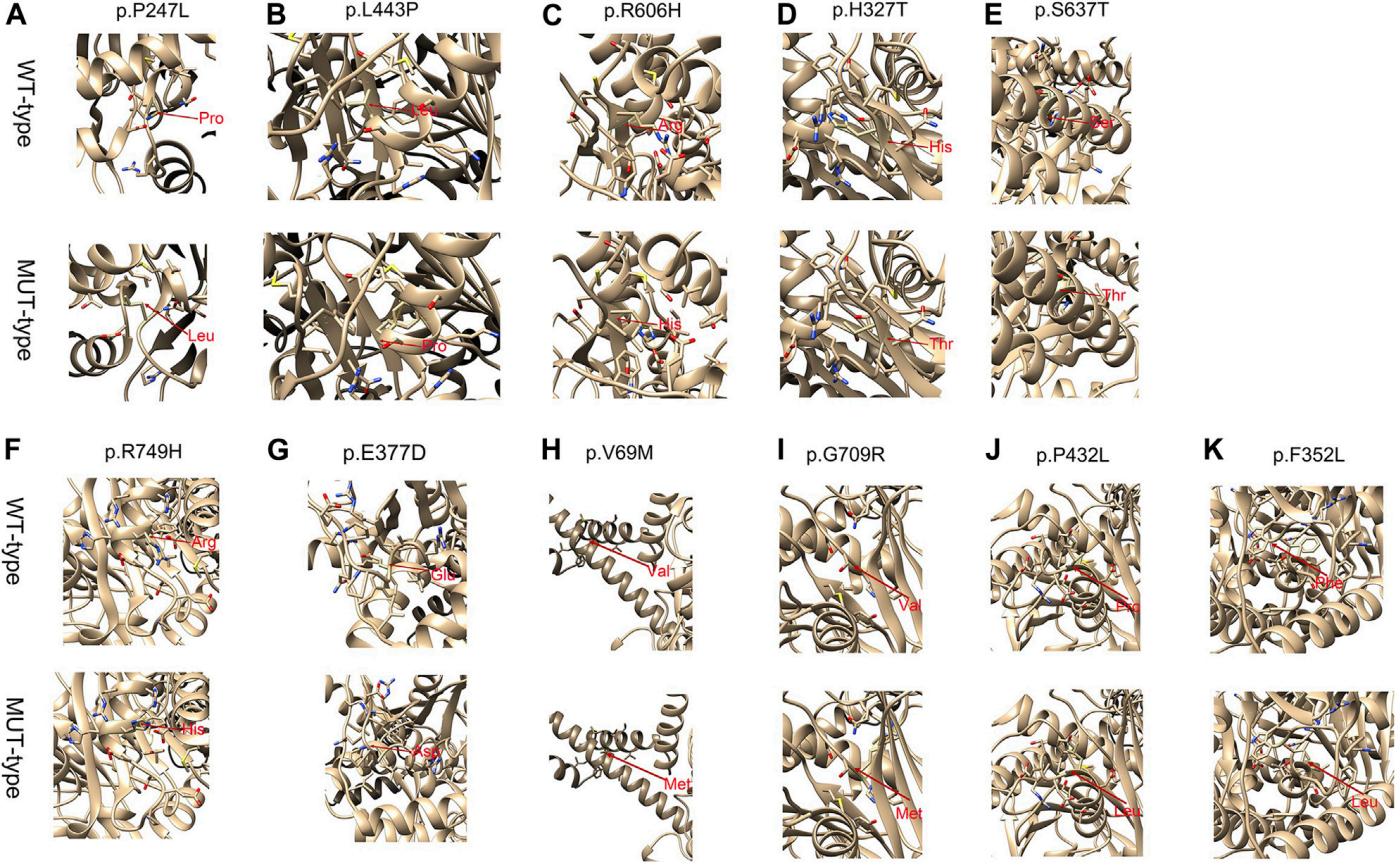
Structure stability analysis of the p.P247L, p.L443P, p.R606H, p.H327T, p.S637T, p.R749H, p.E377A, p.V69M, p.G709R, p.P432L mutations on the partial tertiary structure of CPT1Aprotein (Uniprot: AF-P50416-F1). Structure stability analysis of the p.F352L mutation on the partial tertiary structure of CPT2 protein (Uniprot: AF-P23786-F1). A: The wildtype amino-acid residue at 247 site, 443 site, 606 site, 327 site, 637 site, 749 site, 377 site, 69 site, 709 site, 432 site and 352 site (CPT2 protein) colored green has no contact with the amino-acid residue nearby. **(A)** base on this model, after amino-acid was substituted by the Leucine amino acid residue at this site, five atoms clash and contacts were found in this region. **(B)** After amino-acid was substituted by the Proline amino acid residue at this site, five atoms clash and contacts were found in this region. **(C)** After amino-acid was substituted by the Histidine amino acid residue at this site, three atoms clash and contacts were found in this region. **(D)** After amino-acid was substituted by the Threonine amino acid residue at this site, no atom clash and contacts were found in this region. **(E)** After amino-acid was substituted by the Threonine amino acid residue at this site, one atoms clash and contacts were found in this region. **(F)** After amino-acid was substituted by the Histidine amino acid residue at this site, three atom clash and contacts were found in this region. **(G)** After amino-acid was substituted by the Aspartic acid residue at this site, 0 atoms clash and contacts were found in this region. H After amino-acid was substituted by the Methionine amino acid residue at this site, seven atoms clash and contacts were found in this region. **(I)** After amino-acid was substituted by the Argnine amino acid residue at this site, 37 atoms clash and contacts were found in this region. **(J)** After amino-acid was substituted by the Leucine amino acid residue at this site, 0 atoms clash and contacts were found in this region **(K)** After amino-acid was substituted by the Leucine amino acid residue at this site, one atoms clash and contacts were found in this region. Amino acid change marked with red arrows. Atoms clash and contacts are shown in yellow lines. The Structure stability analysis of novel mutations were performed by Chimera version 1.15.

For all these novel missense variants, all were predicted to be potentially pathogenic byPolyphen-2, PROVEAN, and the MutationTaster predictor (Table 3). Structure stability analysis was performed using Chimera version 1.15. Atoms clash and contacts decreased structure stability. c.740C>T (p.P247L), c.1328T > C (p.L443P), c.1817G>A (p.R606H), c.1910C > A (p.S637T), c.2246G > A, (p.A749H). c.205G > A (p.V69M), c.2125G > A (p.G709R), and (p.P432L). c.1054T > C (p.F352L) might cause 5, 5, 3, 1, 3, 7, 37, and one atom clash and contacts, respectively. No atom clash and contacts were caused by c.1295C > T (p.P432L), c.979C > T (p.H327T), and c.1131G > C (p.G377A). Atoms clash and contact are shown in yellow lines in Figure 1.

**TABLE 3:** Variants detected in confirmed patients with CPT1D, CPT2D, CACTD.

No	Nucleotide	Protein	Location	Effect	Frequency(%)	Prediction of pathogenicity			
						PolyPhen-2	PROVEAN	Mutation taster	References
*CPT1A*									
1	**c.740C**>**T**	**p.P247L**	Exon3	Missense	12.5	D (1)	D (−8.135)	D (0.999)	Novel
2	**c.1328T > C**	**p.L443P**	Exon11	Missense	12.5	D (1)	D (−5.826)	D (0.999)	Novel
3	**c.1817G**>**A**	**p.R606H**	Exon15	Missense	8.33	D (1)	D (−4.557)	D (0.999)	Novel
4	**c.979C > T**	**p.H327T**	Exon10	Missense	4.16	D (0.995)	D (−8.678)	D (0.999)	Novel
5	c.1336G > A	p.G446S	Exon11	Missense	4.16	D (0.995)	D (−5.351)	D (0.999)	Zhang et al. (2021)
6	**c.1910C > A**	**p.S637T**	Exon16	Missense	16.67	D (1)	N (−0.945)	P (0.998)	Novel
7	c.281+1G>A	—	Exon4	Splicing	8.33	N/A	N/A	N/A	Yu et al. (2021)
8	c.956G>T	p.G319V	Exon9	Missense	8.33	B (0.005)	D (−8.203)	D (1)	Yu et al. (2021)
9	**c.2246G > A**	**p.R749H**	Exon19	Missense	8.33	D (1)	N (−0.771)	D (0.999)	Novel
10	**c.1131G > C**	**p.E377D**	Exon10	Missense	8.33	D (0.906)	D (−7.425)	D (0.999)	Novel
11	**c.577delC**	p.M194*	Exon6	Deletion	8.33	N/A	N/A	N/A	Novel
12	**c.205G > A**	**p.V69M**	Exon7	Missense	8.33	D (1)	N (−1.494)	D (0.996)	Novel
13	**c.2125G > A**	**p.G709R**	Exon17	Missense	16.67	D (0.985)	D (−6.766)	D (0.999)	Novel
14	**c.1295C > T**	**p.P432L**	Exon11	Missense	16.67	D (1)	D (−7.054)	D (0.999)	Novel
*CPT2*									
15	**c.988dupT**	p.I332Hfs*2	Exon4	Frameshift	12.5	N/A	N/A	N/A	Novel
16	c.1711C > A	p.P571T	Exon5	Missense	37.5	D (0.985)	D (-6.169)	D (0.999)	Qian J, et al. (2020)
17	c.520G > A	p.G174L	Exon4	Missense	12.5	P (0.997)	N (-0.989)	D (0.999)	T Takahashi et al. (2015)
18	**c.895-896insGGGCAGAGCTC**	p.R303Gfs*6	Exon4	Frameshift	12.5	N/A	N/A	N/A	Novel
19	**c.1054T > C**	**p.F352L**	Exon4	Missense	12.5	B (0.118)	D (-5.229)	D (0.999)	Novel
20	c.1301T > C	p.F434S	Exon4	Missense	12.5	B (0.118)	D (-5.851)	D (0.999)	Nathalie et al. (2021)
*SLC25A20*									
21	c.1A>G	p.M1V	Exon1	start codon	12.5	B (0.028)	N (-1.132)	D (0.999)	Yan et al. (2017)
22	c.199-10T>G	—	Intron2	Intron	62.5	N/A	N/A	N/A	Chen M et al. (2020)
23	c.270delC	p.F91Lfs*37	Exon3	Frameshift	12.5	N/A	N/A	N/A	BB Gürbüz et al. (2021)
24	c.804delG	p.F269Sfs*3	Exon8	Frameshift	12.5	N/A	N/A	N/A	T Fukushima et al. (2013)

Reference sequence for *CPT1A*, *CPT2*, *SLC25A20* are NM_001876.3, NM_000098.2, NM_000098.3 respectively. The previously unreported novel variants of this study are in boldface type. N/A: not available. PolyPhen-2: http://genetics.bwh.harvard.edu/pph2/, HVAR score ranges from 0 (neutral, N) to 1 (damaging, D), B: benign. PROVEAN: http://provean.jcvi.org/index.php, score ranges from ≤ −2.5 (deleterious, D) to > −2.5 (neutral, N). Mutation Taster: http://www.mutationtaster.org/, a score close to one indicates a high “security” of the prediction (P: polymorphism, D: disease causing).

Novel mutations are in boldface type.

## Discussion

In this study, 20 patients with disorders of mitochondrial carnitine–acylcarnitine cycle were identified (12 with CPT1D, 4 with CPT2D, and 4 with CACTD) in approximately 4 million newborns in Zhejiang province, China between 2009 and 2021. The data revealed the largest series of cases with disorders of mitochondrial carnitine–acylcarnitine cycle detected by NBS in the Chinese Population. The incidence of newborns with disorders of mitochondrial carnitine–acylcarnitine cycle was 1:203,518 (CPT1D: 1:339,197; CPT2D: 1:1,017,593; and CACTD: 1:1,017,593) in Zhejiang province. CPT1D was the most common disorder of mitochondrial carnitine–acylcarnitine cycle. The incidence of these disorders in Taiwan was 1:263,523 (CPT1D: 1:790,567 and CPT2D/CACTD: 1:395,283) according to NBS data in 2013 (Chien et al., 2013). Data from the largest nationwide cross-sectional study illustrated that the total incidence in mainland China was 1:236,655 (CPT1D: 1:546,128, CPT2D: 1:1,014,237, and CACTD: 1:709.996) (Deng et al., 2020). Incidence of these disorders in Australia, Germany, and the United States was 1:250,000 to 1:666,667 (Lindner et al., 2010). Disorders of mitochondrial carnitine–acylcarnitine cycle seem to be rare diseases in general population. The incidence of these disorders, especially that of CPT1D, in Zhejiang province seems to be higher than that reported in previous studies. This difference may be due to the awareness of these disorders, ethnic backgrounds, different cut-off values, recall criteria, and development of diagnostic methods. Previous reports revealed that these disorders may present normal acyl-carnitine levels at birth, resulting in false-negative cases (Dowsett et al., 2017). Case 12 with CPT1D was overlooked at the initial screening. Hence, the actual incidence may be higher than the reported data. A looser cut-off value can decrease false-negative cases but increase the false-positive cases. To handle these issues, a previous report revealed that they have developed second-tier molecular tests for these diseases (Lin et al., 2020).

NBS plays an important role in the early detection of deficiency in enzymes of mitochondrial carnitine–acylcarnitine cycle (Wilcken et al., 2003). Most patients with CPT1D presented increased C0 levels and C0/(C16 + C18) ratios and/or decreased long-chain acyl-carnitines levels. For instance, patient 12 with normal C0 levels and C0/(C16 + C18) ratios was overlooked at the initial screening and exhibited slight reduction of C12 to C18:1 level. Milder forms of CPT1D may present normal C0 levels and C0/(C16 + C18) ratios or slight changes can easily be missed by NBS (de Sain-van der Velden et al., 2013; Dowsett et al., 2017). Combining a lower C18:1 level with higher C0/(C16 + C18) ratios in plasma and high sensitivity and specificity for CPT1D could be found in a previous study (Heiner-Fokkema et al., 2017). The reduction in C18:1 level might be an important inducer of CPT1D, which is consistent with a previous study which reported nine patients with a low C18:1 level. The number of patients with low C18:1 level was more than that with low C12 to C16:1 level. Increased C12–C18:1 level (particularly C16 and C18:1) and/or decreased C0, C2 levels were the major characteristics in CACTD and CPT2D. Some patients had normal concentrations of C12, C14, C16, C18, and C18:1. Elevation of (C16 + C18:1)/C2 ratios could reduce the false negative rate and improve the sensitivity (Sigauke et al., 2003; Edmondson et al., 2017; Tajima et al., 2017; Tang et al., 2019). However, this ratio sometimes may be normal (de Sain-van der Velden et al., 2013; Edmondson et al., 2017). The ratio of several long-chain acyl-carnitines to C3 like C14/C3, C16/C3, C18/C3, and (C16 + C18:1)/C3 can be used as better indices for CPT2D screening (Edmondson et al., 2017). In this study, it was observed that C14/C3, C16/C3, C16:1/C3, C18:1/C3, and (C16 + C18:1)/C3 ratios were increased in all patients with CACTD and CPT2D, which were significantly higher in CPT2D; this might be considered to distinguish between these two similar diseases. The results might be caused by very low concentrations of C3 levels in patients with CPT2D.

Patients with CPT1AD presented normal growth and development following a strict diet management and follow-up (Gan et al., 2021). CPT1D can be triggered by fasting or illnesses and presented with hypoketotic hypoglycaemic episodes and hepatomegaly in the early period of life; few cases may die in neonatal period (Baruteau et al., 2014; Fohner et al., 2017). Several cases with mild cardiomegaly, heartbeat disorders, or distal renal tubular acidosis have been reported (Olpin et al., 2001; Yu et al., 2021). Most patients with CPT1D had no clinic symptoms and only one case presented with hyperammonaemia and metabolic acidosis and died in the early neonatal period. Additionally, data suggested that early diagnosis and proper treatment resulted in good prognosis in patients with CPT1D. It remains to be observed whether patients with CPT1D will present further clinical symptoms. Overall, three different phenotypes of CPT2D have myopathic, lethal neonatal, and severe infantile forms. The most common form of CPT2D is the myopathic form, and the symptoms in 70% cases of this form were presented during 0–12 years of ages. The most common clinical symptoms were myalgia, myoglobinuria, and muscle weakness trigged by exercise and infection (Joshi et al., 2014; Joshi and Zierz, 2020). The severe infantile forms always presented hypoketotic hypoglycaemia, hepatomegaly, metabolic acidosis, cardiac manifestations, weakness, seizures, and sudden death (Sigauke et al., 2003). The lethal neonatal form is fatal, which causes death during the neonatal period (Isackson et al., 2008; Du et al., 2017). Overall, 4 CPT2D cases were detected in this study. One lethal neonatal form (patient 14) presented metabolic acidosis and hypoketotic hypoglycaemia and died soon in the neonatal period. One severe infantile form (patient 13) presented sudden death at 8 months at home without any available data. The two remaining cases (patients 15 and 16) were asymptomatic along with incomplete right and transient elevation of ALT, respectively. Critically ill patients with CACTD presented a severe phenotype in the neonatal time, including rapidly progressive condition and a high fatality rate (Chen et al., 2020). Late onset form has been less common (Morris et al., 1998). In this study, the 4 cases with CACTD presented feeding dysfunction, poor response, hypoglycaemia, metabolic acidosis, hepatic impairment, hepatomegaly, and cardiac hypertrophy. In addition, two patients died in the early neonatal period, and 1 case died at 2 months after birth. Patient 17 survived. Although CACT deficiency diagnosed via NBS had a surely high mortality rate, early detection and treatment may save lives.

In general, more than 30 CPT1A pathogenic mutations have been reported (Tsuburaya et al., 2010; Gan et al., 2021). c.1436C > T (p.P479L) was the common mutation in northern Canada, Greenland, Colombia, and the native Alaskan population (Park et al., 2006). Moreover, c.2129G > A (p.G710E) was mainly detected in the US Alaskan and Hutterite populations, and a homozygous mutation might lead to severe clinical manifestations (Prasad et al., 2001; Prip-Buus et al., 2001). Hotspot mutations with CPT1D in the Chinese population have not yet been reported. c.1910C > A (p.S637T), c.740C > T (p.P247L), and c.1328T > C (p.L443P) presented highest occurring mutations in this study and might be the potential hotpot variants in Zhejiang province of South China.

Seventy pathogenic variants in CPT2 gene were reported and phenotype–genotype association was observed in CPT2D (Joshi et al., 2014). c.680C > T (p.P227L), c.1923_1935del, and c.983A > G (p.D328G) were thought to be correlated with lethal neonatal and severe infantile forms and generally not detected in patients with myopathic forms (Isackson et al., 2006; Isackson et al., 2008). Furthermore, the c.338C > T (p.S113L) mutation was detected in almost 70% patients with myopathic forms (Joshi et al., 2014; Fontaine et al., 2018). The c.338C > T (p.S113L) compound heterozygotic patients were easily triggered by low temperature compared with c.338C > T (p.S113L) homozygotic patients. Patients with truncating mutations were significantly easily triggered by fasting compared with those with missense mutations on both two alleges. The c.338C > T (p.S113L) mutation of CPT2 is a common variant in the Caucasian population, whereas c.1148T > A (p.F383Y) mutation is common in Japanese population (Yasuno et al., 2008). According to dataset, the most frequent mutation was c.1711C > A (p.P571T), which was previously reported in the northern China and might be the potential hotspot of CPT2D in Chinese population (Qian et al., 2017).

More than 40 SLC25A20 pathogenic mutations have been identified (Yan et al., 2017). Phenotype–genotype association in CACTD have been reported previously. The c.241G > A (G81R) mutation was associated with severe phenotype, whereas the c.955insC mutation was associated with a milder form in the Netherlands (IJlst et al., 2001). c.199-10T > G mutation was the most common in East Asia, which accounted for 83% of the variant alleles in Chinese population (Tang et al., 2019; Chen et al., 2020). The homozygous c.199-10T > G mutation was correlated with lethal neonatal form in Asian cases; two cases with homozygous c.199-10T > G mutation in our study were died early. c.199-10T > G mutation may cause premature protein truncation and translocate enzyme without activity which may explain the severe genotype of c.199-10T > G mutation (Hsu et al., 2001). A compound heterozygous case with c.199-10T > G and c.1A > G (p.M1V) mutation died at 6 days after birth (Yan et al., 2017), whereas a case in our study with the same mutation survived. Early timely treatment is crucial for CACTD and may reverse the metabolic decompensation. This good prognosis of our case might have benefitted from early detection and early management after NBS.

This study identified 11 novel CPT1A variants and 3 novel CPT2 variants. Deletion (c.577delC (p.M194*)) and frameshift variants (c.988dupT (p.I332Hfs*2) and c.895-896insGGGCAGAGCTC (p.R303Gfs*6)) would result in truncated proteins which would potentially affect protein function. All novel missense variants were predicted as possibly pathogenic by bioinformatics analysis. We further predicted structure stability analysis of novel missense mutations. In addition to c.1295C > T (p.P432L), c.979C > T (p.H327T), and c.1131G > C (p.G377A), all missense variants might cause atom clash and contacts and decreased structure stability of protein resulting in a loss-of-function effect. Nevertheless, the pathogenicity of all these novel variants needs to be confirmed by further functional investigations.

## Conclusion

In conclusion, disorders of mitochondrial carnitine–acylcarnitine cycle, with a rare combined incidence among newborns, were detected through NBS in Zhejiang, China. Most of the patients presented different typical acylcarnitine profiles. To be more specific, most patients with CPT1D presented normal growth and development, whereas those with CPT2D/CACTD had a high mortality rate. Overall, 11 novel CPT1A variants and 3 novel CPT2 variants were identified, which further expanded the mutational spectrum. All novel missense variants were predicted as possibly pathogenic by bioinformatics analysis. This study may help in enhancing overall awareness of disorders of mitochondrial carnitine–acylcarnitine cycle through clarifying the signification of early diagnosis and proper treatment on the prevention and recovery of patients.

## Data Availability

The data presented in the study are deposited in the Figshare. The accession numbers were 10.6084/m9.figshare.18392345 and 10.6084/m9.figshare.18392426.

## Abbreviations

CPT1, carnitine palmitoyltransferase 1; CPT2, carnitine palmitoyltransferase 2; CACT, carnitine-acylcarnitine translocase; CPT1D, carnitine palmitoyltransferase 1 deficiency; CPT2D, carnitine palmitoyltransferase 2 deficiency; CACTD, carnitine-acylcarnitine translocase deficiency; NBS, newborn screening; C0, free carnitine; C2, Acetylcarnitine; C3, Propionylcarnitine; C4, Butyrylcarnitine; C12, Lauroylcarnitine; C14, Myristoylcarnitine; C16, Palmitoylcarnitine; C16:1, Palmitoylcarnitine; C18, Octadecanoylcarnitine; C18:1, Oleylcarnitine; MS/MS, tandem mass spectrometry; DBS, dried blood spot; NGS, next-generation sequencing; PCR, Polymerase chain reaction-restriction.
